# Advancing the Study of “Goals of Best Practice”: Toward Achieving Optimal Best – Educational Implications to Developments in Flow Research and Positive Optimal Psychology

**DOI:** 10.3389/fpsyg.2022.838560

**Published:** 2022-04-11

**Authors:** Huy P. Phan, Bing Hiong Ngu

**Affiliations:** School of Education, University of New England, Armidale, NSW, Australia

**Keywords:** goals of best practice, flow state, meditation, optimal best, flourishing, optimization, mindfulness, personal resolve

## Abstract

The paradigm of *positive psychology* is significant in introducing positive psychological concepts such as “flourishing,” “optimal best,” and “a state of flow.” In terms of research development of positive psychology, the researchers of this article have made extensive theoretical, empirical, and methodological contributions by advancing the study of optimal best. One aspect of this research, notably, consists of advancement of the *psychological process of optimization*. Optimization, in brief, provides a theoretical account into the “optimization” of a person’s state of functioning. Non-academically, a Buddhist nun’s seeking to successfully achieve an optimal state of enlightenment or, academically, a first-year student’s seeking to achieve an A grade in Psych 101 would require some form of optimization. Recent research development has, interestingly, considered a related concept known as “goals of best practice” (GsBP), which may co-exist with the process of optimization and/or assist to account for the optimization of learning experiences. This conceptual analysis article, by utilizing the *paradigm of philosophical psychology*, advances the study of optimal best practice by focusing on three major aspects: (i) to consider conceptually and philosophically how and/or the extent to which GsBP could, in fact, relate to the nature of flow, flourishing, and optimal best; (ii) to consider a methodological account, which could help to measure and assess the concept GsBP; and (iii) to consider the potential practicality of GsBP in educational contexts, which may assist to facilitate and motivate the achievement of optimal best. These three aspects, we firmly believe, are of significance as they provide grounding for implementation and continuing research development into the area of best practice.

## Introduction

The study *flourishing*, coinciding with a “state of flow” (Csíkszentmihályi, 1990, 1997) is an interesting research inquiry in the social sciences, reflecting the nature of the *paradigm of positive psychology* (Seligman and Csíkszentmihályi, 2000; Seligman et al., 2009). Positive psychology, in brief, espouses the importance of remedy and prevention of maladaptive life experiences, and the facilitation and promotion of positive and proactive life experiences. Flourishing (Seligman, 2011; VanderWeele, 2017; Phan et al., 2019b), by reflecting the intricate nature of positive psychology, may consist of and reflect a student’s state of flow and achievement of “optimal best” or “optimal functioning” in subject matter (e.g., Calculus; Fraillon, 2004; Phan et al., 2016). Optimal best in a subject matter, in brief, is defined as the maximization of a person’s internal state of functioning (e.g., cognitive functioning; Phan et al., 2016). Moreover, in accordance with Phan et al.’s (2020c) recent explanation, optimal best is contextualized or is situated within a particular timepoint and domain of functioning. For example, in terms of comparison, optimal best in the domain of physical functioning (e.g., a professional football player’s optimal best in scoring 20 goals in the 2022/2023 season) is different from optimal best in the domain of cognitive functioning (e.g., a secondary school student exceeding in his final Chemistry exam).

**FIGURE 1:**
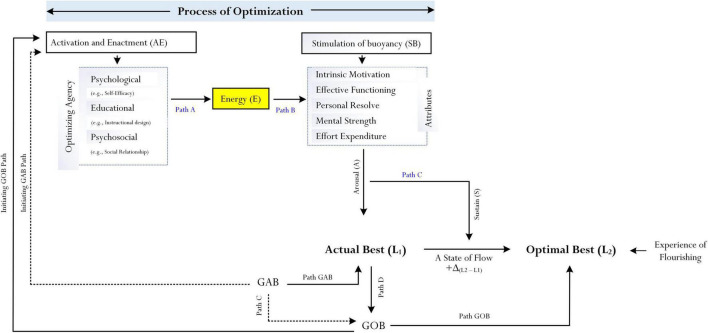
Conceptualization of flow, flourishing, and best practice. Adapted from Phan et al. (2019b).

One aspect of our research development, capitalizing on Fraillon’s (2004) brief introduction of the *psychological process of optimization*, explores the intricate nature of optimal best practice in a specific subject matter. For example, what is it that would cause a 3rd year university student to achieve an optimal state of cognitive functioning in Biology? In a similar vein, does the student experience a state of flow as she engages in her learning and, if so, how does this state of flow relate to personal experience of flourishing? These reflective questions, we contend, have established grounding for our experimental and non-experimental research undertakings, which specifically delve into the “optimization” of optimal learning experience. One interesting line of inquiry, as shown in Figure 1, relates to our conceptualization and personal understanding of the relationships between four interrelated entities: optimization, optimal best, state of flow, and experience of flourishing. Our established findings have so far substantive theoretical and methodological contributions (e.g., Phan et al., 2019b; Phan and Ngu, 2021a) to the study of optimal best (Fraillon, 2004; Phan et al., 2016). For example, in our recent article (Phan et al., 2019b), we discussed and proposed a methodological account that can be used to measure and assess the process of optimization. The focus of this *conceptual analysis* article, consisting of the use of *philosophical psychology* as a methodological paradigm (Thagard, 2014, 2018; Phan et al., 2020b), involves our detailed overview of a proposed concept for consideration, which we term as “goals of best practice” in relation to a particular domain of functioning – say, a Year 11 student’s goal of best practice to achieve an A grade for mathematics. We propose that there are two distinct goals of best practice (GsBP), namely, “goal of actual best” and “goal of optimal best,” which may act as “sources of optimization.” The underlying premise of our conceptualization, specifically, considers an interesting postulation: that GsBP (e.g., goal of optimal best) could serve to initiate and/or to facilitate a person’s flow state and the optimization of individual progress, resulting in their experience of flourishing in a specific subject matter.

## A State of “Positivity”: An Introduction

Proactive *human agency* (Bandura, 1986, 1997) espouses individual choice, autonomy, and freewill. In educational contexts, for example, proactive human agency may consist of a university student choosing to study the subject(s) of their choice. Proactive human agency may relate to a secondary school student’s choice to disengage and withdraw from school altogether and to enroll in vocational training development. In this sense, proactive human agency in educational settings (e.g., a student’s choice to seek mastery in the subject of Buddhist Mindfulness) may instill a sense of motivation, encouraging and fostering a person to strive for optimal learning experiences. In non-educational settings, likewise, proactive human agency may involve the encouragement and promotion of optimal health functioning.

Proactivity of human agency may also intertwine with the positive nature or the “positivity” of life. The positivity of life, in this case, may entail perceived feelings and/or enriched experiences of different types of life qualities (e.g., a person’s perceived feeling of continuing happiness; Phan et al., 2020a). Approaching life from a positive point of view (e.g., seeking to appreciate the meaning of “enlightenment”; Phan et al., 2020b,2021b), in this sense, closely aligns with and reflects the significance of the *paradigm of positive psychology* (Seligman and Csíkszentmihályi, 2000; Seligman et al., 2009). One notable tenet of positive psychology, in this case, emphasizes the importance in cultivation and enrichment of “personal flourishing” of life qualities (e.g., a reflection on inner virtues). Personal flourishing is an interesting life-related concept, which espouses a person’s effective functioning especially in terms of their well-being (Huppert and So, 2013). A person’s testament of “flourishing,” an indication that life is going well (e.g., “I feel pretty good at the moment…”) may reflect their positive mental well-being (Keyes, 2002; Keyes et al., 2002). Academically, for example, a secondary school student’s a state of flourishing may reflect on his feeling of happiness as he engages socially with other students.

The proactivity of human agency also emphasizes the importance of another related concept known as a “state of flow” or a “flow state.” A state of flow (Csíkszentmihályi, 1990, 2014a,b) is positive and may reflect a person’s absorption and engagement with a particular course of action. Flow is positive and coincides with the analogy of a “positive current.” which may indicate a person’s experience of “energy,” “excitement,” etc. For example, an adolescent may experience a state of flow when he plays Doom Eternal^1^ with his two best friends, whereas a senior citizen may experience a state of flow as she practices Buddhist meditation. Academically, say, a university student’s appreciation and enjoyment of Calculus may reflect a state of flow, reflecting her strong commitment to seek deep and meaningful understanding despite some minor setbacks. A point of commonality between the adolescent, the senior citizen, and the university student is that there is a perceived feeling of absorption, entrenchment, enjoyment, and appreciation by which contextual influences (e.g., another commitment or event) do not make a difference and/or profound impact. Having said this, however, we acknowledge the fact that there may be personal “contexts” which could determine and/or account for how flow is experienced. For instance, a student may need to have personal conviction, self-belief, determination, affliction, etc. for what they do, and this in turn might contribute to the experience of flow.

A state of flow is not instantaneous but rather functions within a dynamic system of personal change. As a person progresses through a course of action, she experiences two interrelated aspects, which are positive, proactive, and motivational: underlying process vs. accomplished outcome. For example, within the context of secondary school learning, a Year 10 student may wish to seek meaningful understanding and mastery of Scandinavian furniture making.^2^ This personal intent is positive and, importantly, would entail the following:

(1.) The student’s positive experience of a learning process, which may reflect his feeling of contention, enjoyment, and satisfaction.
(2.) The student’s feeling of personal accomplishment, which may reflect his eventual mastery in furniture making.

The above description emphasizes the importance in perceived positivity of a contextual nature of a situation, task, event, etc. at hand (e.g., an adolescent and her best friend enjoying a musical concert). Perceived negativity of an unfavorable situation, task, event, etc. (e.g., a struggling student having to understand the basics of Calculus for a mid-year exam) would constrain a state of flow and, in contrast, instill the personal feeling and experience of procrastination, helplessness, pessimism, etc. This testament, acknowledging a distinction between perceived positivity and perceived negativity, rationalizes a continuous learning process, which espouses contrasting motivational states (e.g., a state of flow *versus* a state of procrastination) and accomplished outcomes.

There is credence from existing findings (e.g., Engeser, 2012; Schiepe-Tiska and Engeser, 2012; Harmat et al., 2016; Csíkszentmihályi and Nakamura, 2018; Rutrecht et al., 2021; Tse et al., 2021; van der Linden et al., 2021) to support the study of flow in different contexts. As the literature shows, one interesting aspect of research development has focused on the neuroscience of flow states (e.g., Gold and Ciorciari, 2020; Ottiger et al., 2021; van der Linden et al., 2021). van der Linden et al.’s (2021) recent mini review, for example, is extremely insightful, providing theoretical understanding into the extent to which the brain’s locus coeruleus-norepinephrine (LC-NE) system could account for a wide range of behavioral and subjective manifestations of flow. From the perspective of academia and schooling, in particular, a state of flow, similar to that of motivation (Gottfried, 1985; McClelland, 1987; Maehr, 1989; Franken, 2007), may help to account for a person’s improvement, progress, and/or development of different types of adaptive outcomes (e.g., a student’s reporting of her experience of flourishing in learning Calculus). Our research interest in the advancement of flow, as discussed in this article, concerns a conceptual analysis by which we make an attempt to situate a state of flow within the framework of best practice (Fraillon, 2004; Phan et al., 2016).

## Achievement of Optimal Best

Flow is intimately linked to a state of best practice. *Best practice* is a term which, in part, arises from Fraillon’s (2004) seminal policy publication and delves into a person’s theoretical understanding, experience, and/or performance of a specific domain of functioning. According to Fraillon (2004), there are two “levels” of best practice: *actual best* level, denoted as L_1_, and *notional best* level, denoted as L_2_. In conjunction with the two levels of best practice, Fraillon (2004) also introduced a term known as “optimization,” which subsequently led to our expansion and proposition of a theory that we coined as the “Framework of Achievement Bests” (Phan et al., 2017). According to Fraillon’s (2004) brief theoretical account, optimization is a process that could link the two levels of best practice, L_1_ and L_2_, into one relationship. We recently refined the Framework of Achievement Bests theory to include some additional aspects for consideration (Phan et al., 2019b,2020c). For example, in our refinement of the Framework of Achievement Bests (e.g., Phan et al., 2019b; Phan and Ngu, 2019), we theorize that the analogy of the process of optimization is one of “water flowing bursting through a water pump or water hose.”

Importantly, our refinement of the Framework of Achievement Bests (e.g., Phan et al., 2019b; Phan and Ngu, 2019) contends that optimization, as a psychological process, does not equate to a correlation (e.g., Variable A ↔ Variable B, where “↔” = correlation) and/or a prediction of one variable onto another (e.g., Variable A → Variable B, “→” = prediction). Moreover, our theoretical account postulates a variable, which we term as an “optimizing effect,” denoted as “γ” (Phan et al., 2019b). An optimizing effect, or γ, is different from a correlation (i.e., *r*) and/or a predictive effect (i.e., β) and connotes, from our point of view, some form of “force” or “energy.” This force or energy, in turn, would act to “energize” and/or motivate a person to achieve an optimal state of functioning (Phan et al., 2019b,2020c).

The present article, consolidating existing research development, uses *philosophical psychology* (Thagard, 2014, 2018; Phan et al., 2020b) as a methodological basis to develop a conceptual analysis, which may advance the study of optimal best. Philosophical psychology is a methodological paradigm that emphasizes the importance of a person’s intuition, logical reasoning, and ideas, and their use of prior research inquiries to conceptualize and propose theoretical concepts and/or associations between theoretical concepts. This conceptual model, as shown in Figure 1, acknowledges the possibility that a state of flow, personal experience of flourishing, and achievement of best practice could co-exist and interrelate with each other within a “positive system” of change. Moreover, in tandem with our current research focus (Phan et al., in press), we also take this opportunity to consider another aspect of best practice, which forms the basis of this article: the notion of what we term as “goals of best practice.”

### Flow, Flourishing, and Best Practice

A “positive system of change,” from our point of view, contends a constant flux of “movements,” which could produce both educational and non-educational yields. Our conceptualization, as shown in Figure 1, depicts an ongoing dynamic system by which a state of flow (Csíkszentmihályi, 1990, 2014a,2014b) would assist to facilitate the achievement of optimal best or the successful experience of flourishing in a particular domain of functioning. What are some examples of successful optimal best? Some examples include the following (Phan et al., 2020a): (i) academic learning, for example, a student’s optimal cognitive functioning in essay composition where he is able to write a 5,000-word essay and subsequently receiving an A + grade; (ii) personal well-being in a workplace environment, for example, a bank employee’s optimal state of resilience, personal resolve, and motivation to overcome difficulties and achieving exceptional KPIs; (iii) health functioning on a daily basis, for example, a senior citizen’s optimal state of health despite her recent temporary illness from the COVID-19 pandemic; and (iv) professional sports performance (e.g., European football), for example, a football player’s optimal physical and creative ability to score 50 goals in the 2020/2021 season.

Successful accomplishment of optimal best, from our theorization (Phan et al., 2019b,2020c), requires some form of “propellation” or propulsion. This notion of propellation suggests, perhaps, the enactment of some source of force and/or energy. In this analysis, it is plausible to consider a state of flow as a source of force or a source of energy, which then would optimize or “propel” a state of functioning from Time 1 to Time 2. In other words, aside from the enactment of optimization, we contend that a state of flow would facilitate a person’s successful accomplishment of L_2_ from L_1_. Academically, for example, a state of flow may serve to assist and/or to optimize and motivate a secondary school student to progress from knowing how to solve equations with one unknown, *x* (i.e., her accomplishment of L_1_), to simultaneous equations with two unknowns, *x* and *y* (i.e., her accomplishment of L_2_). Non-academically, likewise, a state of flow may motivate and propel a professional football player to improve his scoring 15 goals for the 2019/2020 season (i.e., L_1_) to 25 goals for the 2020/2021 season (i.e., L_2_).

Differing somewhat from previous conceptualizations (Csíkszentmihályi, 1990, 1997, 2014b), which delve into the intricate nature of the task itself (e.g., the enticing nature of a learning or non-learning task, which could result in a person’s state of flow), we consider the relevance and applicability of actual best practice, L_1_, as a point of reference for the stimulation of flow. We postulate that actual best practice in a subject matter (e.g., how much do I know at this stage?) could serve as a reference point (e.g., the placement of a numerical value of, say, “+ 1” for L_1_) by which a person uses to motivate and propel themselves to strive and achieve optimal best (i.e., L_2_). How would we entice and/or motivate a state of flow, especially when the contextual matter at hand is “non-enticing” (e.g., the learning of the topic Differential Calculus)? Our recent conceptual analysis article, for example, considers the use of different types of verbal discourse (e.g., the use of encouraging feedback) to help facilitate the instilment of flow (Phan et al., in press).

The present article, in tandem with Phan et al.’ (in press) recent conceptual analysis article, considers an interesting theoretical concept known as “goals of best practice,” which refers to personal goals that a person may anticipate and set. The underlying premise of our conceptualization, as detailed in the next section of this article, relates to the possibility in which GsBP (e.g., “My goal for this semester is to ensure that I master deep, meaningful understanding of Psych 101”) could serve as an “instigator” of a state of flow. This conceptualization (i.e., GsBP → a state of flow) interestingly places emphasis on the instilment of personal commitment, personal resolve, self-confidence, and motivation, which could function as potent sources of information in the formation of flow.

## Conceptualization: Theory of Goals of Best Practice

Achieving optimal best, from existing research development, requires some form of initiation and/or assistance. From the preceding sections, it is plausible to consider a state of flow (Csíkszentmihályi, 1990, 1997) as a source of initiation, which could assist to facilitate a person’s achievement of L_2_. To advance this point (i.e., the optimization of achievement of L_2_ via different means, such as experience of flow), we recently conceptualized a theoretical concept known as “goals of best practice,” abbreviated as “GsBP,” which could potentially play a prominent role in helping to initiate and/or to optimize a person’s achievement of L_2_. Our conceptualization, as shown in Figure 1, considers two main goal types: “goal of actual best” (denoted as “GAB”) and “goal of optimal best” (denoted as “GOB”). We rationalize that the nature of GAB is different somewhat from the nature of GOB, which is positive, proactive, and motivational. A specific GAB, we contend, may act a point of reference, which then would inform and assist a person to consider their GOB. In this analysis, successful experience and accomplishment of L_1_ that corresponds to a specific GAB would serve to guide and direct a person towards the setting of a corresponding GOB. From this description, we contend that there is a close correspondence between GAB and L_1_ and between GOB and L_2_. Particularly, a specific GAB would result in a person’s experience and successful accomplishment of L_1_ (i.e., Path GAB), whereas a GOB would result in their experience and successful accomplishment of L_2_ (i.e., Path GOB). In essence, this rationalization considers that both goals of actual best (i.e., denoted as “GsAB”) and optimal best (i.e., denoted as “GsOB”) could account for and/or facilitate a person’s state of flow, or + Δ_(L2 – L1)_. There are different theories of personal goals – for example, *theory of personal best goals* (Martin, 2006; Martin and Liem, 2010; Liem et al., 2012) and *theory of achievement goals* (Ames and Archer, 1988; Elliot et al., 2011; Van Yperen et al., 2014). Our theory of GsBP is somewhat different, as it focuses on the contextual nature of best practice – namely, L_1_ and L_2_ (e.g., a secondary school student’s personal goal of achieving a state of L_2_ in Calculus). Moreover, our focus of inquiry, forming the premise of our conceptualization, concerns the extent to which personal goals that individuals anticipate, construct, and/or set would coincide with and/or support the operational nature of optimization. In this analysis, as shown in Figure 1, personal goals may assist, facilitate, and/or account for a person’s experience of flow state, which, in this case, equates to a positive difference between L_1_ and L_2_. This theoretical premise is significant, suggesting that perhaps the setting of personal goals of different types would assist to facilitate personal experience of absorption, intense concentration, enjoyment, and vigor during the course of an action.

### Goal of Actual Best: A Proposition

*Goal of actual best*, GAB, is defined as “an individualized personal goal that a person may indicate, reflecting his/her intent and conviction to remain on task with a particular course of action without any desire and/or aspiration for achievement of optimal best.” This definition contends that a person’s specific GAB would coincide with and/or associate with their state of L_1_. At present, say, what does a secondary school student indicate and/or attest to in terms of their state of L_1_? At a particular point in time, a person may construct and/or set a GAB, which would coincide with their current experience of L_1_ in a specific subject matter in terms of knowledge, understanding, skills, etc. (e.g., “My GAB, at the present time, is to fulfil what is being asked of me in Chemistry”).

Goals of actual best are specific, situated to a particular time context and domain of functioning. Academically, for example, a first-year university student may construct and set a specific “GAB” where she intends to complete a 2,000-word essay on Sigmund Freud’s (1920) *psychosexual theory of personality* and, in the process, receiving a C grade for her time and effort. Non-academically, an employee of Citibank may set a GAB, which showcases his intent to recruit 2–3 customers for personal bank loans for the forthcoming month. Moreover, from these examples, we posit that GsAB are time-specific to the time context where everything “is here and now.” It is not, in contrast, an anticipation, construction, and/or setting of a personal goal of what a person could accomplish *in future* (e.g., “My GAB, two months from today, is to fulfill….”).

Goals of actual best, from our point of view, are *actual* and *realistic*. On a daily basis, in this sense, a specific GAB would indicate and reflect a person’s current intent to remain on course without any consideration for change for the better. Individually and subjectively, of course, GsAB differ between individuals and for different contexts. For example, academically, one student’s GAB may indicate his intent to complete a 2,000-word essay and receiving a C or B grade for this effort, whereas another student’s GAB may reflect her intent to remain on course with a unit of study and receiving an A^+^ grade. Moreover, in accordance with our theorization, the nature of GsAB does not allude to any indication and/or evidence of aspiration of intent to progress for the purpose of achieving optimal best, i.e., L_2_.

### Goal of Optimal Best: A Proposition

*Goal of optimal best*, GOB, is defined as “an individualized goal that a person may indicate, reflecting his/her conviction and aspiration of intent to strive and achieve optimal best in a subject matter.” This definition contends that a specific GOB would coincide with and/or associate with a state of L_2_. At a particular point in time, a person may set a GOB which would coincide with their state of L_2_ in a particular domain of functioning in terms of knowledge, understanding, skills, etc. (e.g., “My GOB is to consolidate what I know so far and, as a result, to strive for optimal learning experiences in Chemistry”).

Similar to GsAB, GsOBs are specific, situated to a particular time context and domain of functioning. Academically, for example, a secondary school student may set an “GOB” where he aspires with the intention to achieve an A grade for Mathematics. Non-academically, a bank employee may set a specific GOB, which showcases her aspiration of intent to achieve a promotion by the end of the year. Differently, however, we posit that GsOB are time-specific to a known or an unknown *timeframe into the future*, which one may require in order to successfully accomplish the designated goal or goals. How long, for example, would it take a secondary school student to achieve a personal goal (i.e., GOB) of receiving an A grade for Chemistry? One school term? Six months? A year from now? In a similar vein, how long would it take a bank employee to achieve a promotion? Is it feasible and/or achievable with a six-month window?

Goals of optimal best are purposeful and aspirational, reflecting a person’s desire and intent to flourish in a subject matter. The nature of GsOB, in contrast to GsAB, is positive and motivational, serving to govern and to direct a person to strive for achievement of optimal best. A specific GOB, from our conceptualization, is subjective and contextual, aligning to a particular domain of functioning. A university student’s GOB to achieve an A grade for Psych 101 does not necessarily relate to their GOB to achieve an A grade for Statistics 101. Distinctively, however, our conceptualization rationalizes that GsOB are *realistic* and *attainable*, coinciding with a person’s existing repertoire of knowledge, skills, understanding, etc. In other words, from this rationalization, existing understanding, knowledge, skills, etc. would act to govern a person’s decision in the construction of a GOB or GsOB. For example, it would be unrealistic and/or unattainable for a university student who is struggling, academically, to set a suite of GsOB, which reflect her aspiration of intent to graduate with *cum laude*.

### Interrelatedness Between Goal of Actual Best and Goal of Optimal Best

**FIGURE 2:**
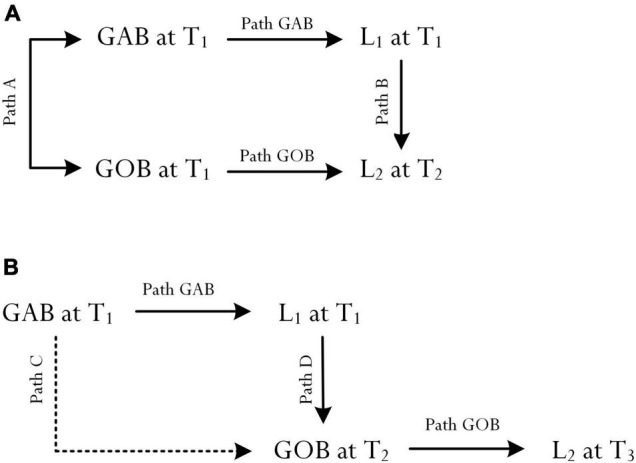
Association between goal of actual best (GAB) and goal of optimal best (GOB). L1 = Actual best, L2 = Optimal best, T1 = Time 1, T2 = Time 2, T3 = Time 3, GAB = Goal of actual best, and GOB = Goal of optimal best. **(A)** shows both GAB and GOB situated at T_1_ (i.e., correlation between GAB and GOB) and **(B)** shows that GAB at T_1_ precedes GOB at T_2_.

The preceding section has provided a brief theoretical account of GsBP. We reason and speculate that GsAB and GsOB may intimately relate to each other, reflecting timely progression and potential individual growth. It is plausible of course, as shown in Figure 2A, that a person may set both types of goal practice at a particular time point (e.g., 4 October 2021) – for example: “My actual best goal, at the present time, is to fulfil what is being asked of me from Mrs. Fergate” and “My optimal best goal is to consolidate what I know so far and, as a result, to strive for an optimal level so that Mrs. Fergate will know…” In this analysis, aside from the potential positive association between the two goal types (i.e., Path A), we postulate that: (i) GAB at Time 1 would positively influence L_1_ at Time 1 (i.e., Path GAB), (ii) GOB at Time 1 would positively influence L_2_ at Time 2 (i.e., Path GOB), and (iii) L_1_ at Time 1 would act as a source of L_2_ at Time 2 (i.e., Path B).

It is plausible, as shown in Figure 2B, for us to consider an additional or an alternative conceptualization in which there is a time displacement between the setting of GAB at Time 1 and the setting of GOB at Time 2. This time displacement, from our point of view, considers the fact that personal contemplation, time, effort, different sources of motivation, etc. would assist with the setting of GsOB. In other words, GsOBs are not instantaneous and/or spontaneous and, from this theoretical account, they do not coincide with the setting of GsAB at a particular time point. For example, within the context of academic learning, personal experience of L_1_ at Time 1 could act as a source of motivation, informing a student on their setting of GsOB at a later date (i.e., Path D). In a similar vein, the setting of GsAB at Time 1 may also assist the student to focus on the setting of GsOB at Time 2 (i.e., Path C), which then would facilitate in the achievement of L_2_ at Time 3 (i.e., Path GOB).

The notion of time in itself, inferring a time displacement between events, situations, experiences, etc. is an interesting concept for consideration. We recently wrote an article, entitled “*Future time perspective and the achievement of optimal best*” (Phan et al., 2020c), in which we explored the nature of time and, in particular, the theoretical concept of *future time perspective*, commonly known as FTP (Wallace, 1956; Kastenbaum, 1961; Nuttin, 1964; Mehta et al., 1972). Nuttin’s (1964) description of time, emphasizing a “future time point” is extremely insightful and it suggests that time is linear – “A simple analysis of human behaviors calls attention to the fact that man [and woman], in his [/her] dealing with a given situation, is usually directed toward something which is not yet there, something which is still to come, something different, even something new [.], are all oriented toward something ahead, something that they are looking for: their behavior is ‘future bound’[.]” (p. 60). We appreciate the significance of time, especially in relation to the study of the aforementioned variables and their relationships (e.g., Figure 1). Our underlying premise, concurring in part with recent research development into the neuroscience of time and flow (e.g., Wittmann, 2011; Rutrecht et al., 2021), contends that time displacement could indeed function as a confounding factor, which then would account for a state of flow, personal experience of flourishing, and/or the operational mechanism of optimization (Phan et al., 2019b). Interestingly, in a recent study, Rutrecht et al. (2021) found that time that time perception is intricately linked to flow states. Particularly, that the more flow someone experiences, the less they think about time, and the faster time passes for them.

Research development pertaining to the neuroscience of flow and time is significant, providing evidence and theoretical insights which may concur with and/or assist us in our conceptualization of relationships between GsBP, levels of best practice, flow states, and personal experience of flourishing (e.g., Figures 1, 2). For example, conceptually, it is of interest for us to consider whether and/or to what extent a person’s inner time experience of flow (e.g., at Time 1) could account for and/or explain their achievement of L_2_. In a similar vein, it is logical to suggest that time experience and accomplishment of L_1_, while experiencing a state of flow, could influence a person’s reaction time and their subjective perception of inner time experience. In relation to Figure 2, Path A and Path C are interesting as they indicate two contrasting conceptualizations: a close association, *r*, between the two goal types vs. GAB acting as a source of GOB. This distinction, we contend, is noteworthy for future research development in terms of empirical validation. Path C (i.e., T_1_ GAB → T_2_ GOB), for example, is logical as it supports our appreciation of time – that time displacement is actually needed in order for a person to set a specific GOB or a suite of optimal best goals for future accomplishment. Moreover, differing from Path A, Path C acknowledges the important fact that GsAB are framed within the context of “here and now” (e.g., T_1_ GAB → T_1_L_1_), whereas GsOB are future focused. This postulation is interesting as it reflects a proposed sequencing between the setting of GsAB and GsOB.

### Goals of Best Practice and Optimization

One aspect of our research development, as reflected in Figure 1, relates to the potential operational nature of GsBP within the framework of optimization (Phan et al., 2017, 2019b,2020c). Specifically, in terms of schooling and academic learning, the “optimization of personal learning experience” is a positive endeavor for accomplishment. How does an educator in a classroom context assist to optimize a student’s academic learning experience of Calculus? This reflective question, which to date has received minimal attention, emphasizes the importance of what we term as “initiators” and/or “activators” of the optimization (e.g., Fraillon, 2004; Phan et al., 2017, 2019b). In the context of personal best or best practice, an initiator and/or an activator is some unknown variable(s), or factor(s), that would “trigger” the positive enactment of the process of optimization (Phan et al., 2019b). What is it that would initiate and/or activate an optimizing agent for change, such as personal self-efficacy for academic learning (i.e., psychological agent; Bandura, 1977, 1997)? In a similar vein, non-academically, how would we initiate and/or activate the optimization of a professional football player’s capability?

Using philosophical psychology as a methodological paradigm (Thagard, 2014, 2018; Phan et al., 2020b), we have explored and have proposed a theoretical model of best practice (Phan et al., 2017, 2019b) which may account for and/or explain the optimization of a person’s state of functioning. As a recap, in this case, the notion of an energizing force from “within” is central to the optimization of, say, a university student’s learning experience from Time 1 to Time 2. Energy, reflecting a perceived sense of propulsion, is crucial, helping to propel a person’s effort, time, action, and motivation to strive for a state of L_2_ (Phan et al., 2019b,2021a). Having said this, conceptually, is it plausible to embrace and/or to incorporate a person’s GsBP as “sources of optimization” or “initiators of optimization”?

We consider, in this case, a conceptualization by which the setting of a specific GOB [e.g., a university student’s personal goal (i.e., GOB) to achieve an A grade for Psych 101 for Semester 1] would operate in a backward loop (i.e., Initiating GOB Path in Figure 1) to initiate the enactment of one or more of the proposed optimizing agents (e.g., psychological agent: self-efficacy, Bandura, 1997) of the process of optimization. This conceptualization of a backward loop (e.g., GOB → activating a person’s inclination to consider her self-efficacy beliefs for academic learning), which, in this case, operates as a psychological agent importantly addresses one fundamental shortcoming of previous theorizations of optimization (e.g., Fraillon, 2004; Phan et al., 2017, 2019b), namely, that an explanatory account into the initiation and activation of optimizing agents and, hence, the totality of the process of optimization has not been explored. In a classroom context, for example, what would initiate and/or activate the “optimization” of a student’s maximized learning experience for Algebra? We could, in this sense, consider other confounding factors that could assist to initiate and/or activate the optimization of a student’s optimal learning experience of Algebra – say, *personal interest* (i.e., personal interest in Algebra learning), *perceived value* of the subject matter itself, *intellectual curiosity*, *philosophical belief*, etc.

The underlying premise of our conceptualization, in this regard, contends that anticipation, construction, and/or setting of personal GsBP could help to initiate and/or activate the process of optimization. As shown in Figure 1, we posit that GsOB (i.e., Initiating GOB Path in Figure 1) and, to a lesser extent, GsAB (i.e., Initiating GAB Path in Figure 1) could act as “initiators” and/or “activators” of the process of optimization. Having said this, however, in terms of a comparison, we argue that GsOB would have more “explanatory power” than GsAB in the optimization of a person’s functioning (i.e., Initiating GOB Path > Initiating GAB Path in the optimization of learning experience of Calculus). The proposed dynamics between the two goal types could, likewise, account for the potential operational functioning of GsAB (e.g., Path C in Figure 1, which shows the positive effect of GAB on GOB).

One interesting aspect of our conceptualization considers the operational nature of GsOB, which could account for and/or explain a person’s flow state (Csíkszentmihályi, 1997, 2014b; Engeser, 2012). In the context of best practice (Fraillon, 2004; Phan et al., 2016) and the process of optimization (Phan et al., 2017, 2019b), we rationalize and contend that a state of flow is equivalent to a person’s successful achievement of L_2_ from L_1_. In other words, a state of flow in a specific subject matter is equivalent or analogous to the notation of “+ Δ_(L2 – L1)_,” which means a positive quantitative and/or qualitative difference between L_1_ and L_2_. Reflectively, how would we facilitate personal experience of flow or, in this case, a quantitative and/or qualitative difference between two levels of best practice? Our theoretical premise, as detailed in Figure 1, contends that the operational nature of a GOB or GsOB would direct, guide, and/or motivate a person to strive for achievement of L_2_. Successful accomplishment of L_2_ from L_1_ in a particular domain of functioning would, in turn, affirm an improvement, progression, and/or experience of a flow state. This proposition (e.g., GOB → L_2_, resulting in improvement, progression, etc.), importantly, also helps to explain the relationship between a flow state and optimization in that, in part or largely, optimization is needed to assist in the facilitation of a flow state.

### Magnitude of Complexity

One major difference between the two GsBP, which may discern their distinctive nature and characteristics, relates to what is known as the “magnitude of complexity.” The magnitude of complexity of a GBP, we contend, would assist to differentiate and affirm as to whether it is a GAB or a GOB. Importantly however, in conjunction with the theoretical tenet of optimization (Phan et al., 2019b), we postulate that the “magnitude” of a GOB, when compared with a GAB, would closely associate with the strength of the process of optimization (i.e., how much optimization would be needed to ensure that a person achieves a state of flow, or their successful experience of L_2_; Phan et al., 2019b).

Perceived complexity of a GBP within the context of academic learning may espouse the notion of “cognitive complexity.” For example, the problem of “Solve for *x* and *y* of *x* + 2*y* = 10 and 4*x* – *y* = -5” is more complex than that of “Solve for *x* of *x* + 5 = -10.” On the other hand, students’ GsOB, on a daily basis, differ in terms of perceived cognitive complexity. For example, in relation to university studies, we may observe the following: (i) the complexity of a GOB, which may consist of a first-year student’s aspiration and intention to achieve a B grade for Psych 101 (Case 1); and (ii) the complexity of a GOB, which may consist of a first-year student’s aspiration and intention to achieve an A grade for Psych 101 (Case 2). A comparison of the two cases shows that the GOB for the student in Case 2 is more complex, reflecting her aspiration, ambition, and motivation to strive for exceptionality.

**FIGURE 3:**
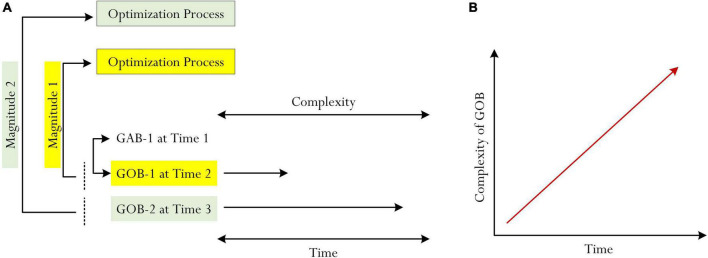
Complexity of GOB and the magnitude of optimization. **(A)** shows the magnitude of the process of optimization and **(B)** shows the complexity of GOB.

Magnitude of complexity, we contend, may play a prominent role in helping to identify individual differences of GOB. Importantly, of course, stipulation of a GOB reflecting a particular level of complexity (e.g., aspiration of intent to achieve an A grade *versus* aspiration of intent to achieve a B grade) is likely to coincide with the process of optimization (Phan et al., 2017, 2019b). In this analysis, we theorize that the magnitude or strength of optimization (e.g., how much optimization would be needed to […]?) would, in part, depend on and/or associate with the complexity of a GOB. Consider Figure 3A, which shows a visual conceptualization of the potential association between the magnitude or strength of optimization and the complexity of GOB. A GOB that is relatively simple in terms of complexity (e.g., GOB-1 at Time 2: a university student’s GOB to achieve a B grade for Psych 101) correspondingly reflects a modest level of aspiration with a low-modest level of optimization (i.e., Magnitude 1). In contrast, however, a GOB that is complex (e.g., OBG-2 at Time 3: a university student’s OBG to achieve an A grade for Psych 101) would require a higher level of optimization (i.e., Magnitude 2).

Changes in strength, or magnitude, of optimization (e.g., a low level of optimization vs. a high level of optimization) are intricately linked to the differential use of time, effort, resources, etc. For example, in relation to the above example, a student who aspires with a personal intent to achieve a B grade (i.e., GOB-1 from Figure 3A) would spend a modest amount of time and effort, utilizing 1–2 instructional approaches (i.e., resources). Aspiring and intending to achieve an A grade, in contrast, would compel a student to expend more effort, more resources (e.g., the use of 2–3 instructional approaches), etc.

As shown in Figure 3 (i.e., Figures 3A,B), we propose an interesting concept that may intimately associate with the magnitude of optimization and the complexity of GOB: *time*. This conceptualization of time coincides with a recent publication (Phan et al., 2020c), which references the relationship between cognitive complexity of optimal best and time difference (Phan et al., 2020c). According to Phan et al. (2020c), the optimal best of a subject matter that is cognitively complex (e.g., a university student’s achievement of optimal best of an A grade in Psych 101) would require more time to undertake and successfully complete (e.g., 3 months). By the same token, from Phan et al.’s (2020c) rationalization, optimal best that is simple and more easy to achieve would require less time (e.g., 3 weeks).

We propose a similar rationalization where we believe there is a close association between the following: perceived cognitive complexity of GOB, the magnitude of optimization, and time (e.g., Figures 1, 3). In this analysis, considering Phan et al.’s (2020c) recent theoretical account, we contend that time in itself is a central variable, or aspect, which could influence a person’s setting of a specific GOB and, in the process, assist in the achievement of optimal best. For example, the “tightness” of time could influence and/or compel a first-year university student to consider a less complex GOB for accomplishment (e.g., a student’s aspiration of intent to achieve a C grade for Economics/Finance 101). The availability of time (e.g., 3 months as opposed to, say, 3 weeks), in this instance, would enable and/or provide continuing optimization, allowing a person to consider more complex GsOB. Thus, as shown in Figure 3A, we consider the following stipulation:

• *The restriction of time*: the limitation of optimization, resulting in the setting of less complex GsOB.
• *The availability of time*: the opportunity and/or provision of optimization, resulting in the setting of more complex GsOB.

## Methodological Development for Consideration

*Philosophical psychology* is an interesting methodological paradigm which emphasizes the importance of personal intuition, theoretical understanding, and logical reasoning (Thagard, 2014, 2018; Phan et al., 2020b). In recent years, researchers have actively used philosophical psychology to assist with the seeking of new frontiers in knowledge and theoretical understanding. For example, one prominent theoretical orientation that we and other researchers have explored and advanced is that of the psychological process of optimization (Fraillon, 2004; Phan et al., 2017, 2019b) which may intricately associate with the nature of optimal best (Fraillon, 2004; Phan et al., 2016). Recognizing the importance of this conceptualization, likewise, in related research in 2020 which also used philosophical psychology, has considered the potentiality for *Buddhist mindfulness* (Nyanaponika Thera, 1972; Hanh, 1976; Yeshe and Rinpoche, 1976) to co-exist with and/or to situate within the framework of optimization (Phan et al., 2020b).

Advancing the study of optimization (Fraillon, 2004; Phan et al., 2017, 2019b), we consider a related concept of GsBP, which may closely associate with a state of flow, the personal experience of flourishing, and the process of optimization. Our rationalization to include GsBP is significant as this theoretical concept is positive and motivational, guiding a person to strive for optimal best. So far, however, our conceptualization of GsBP is philosophical with a clear lack of empirical evidence for support. As such, we suggest a priority in research focus, empirically and/or methodologically, which may support our philosophical theorization (e.g., Figures 1, 3).

**FIGURE 4:**
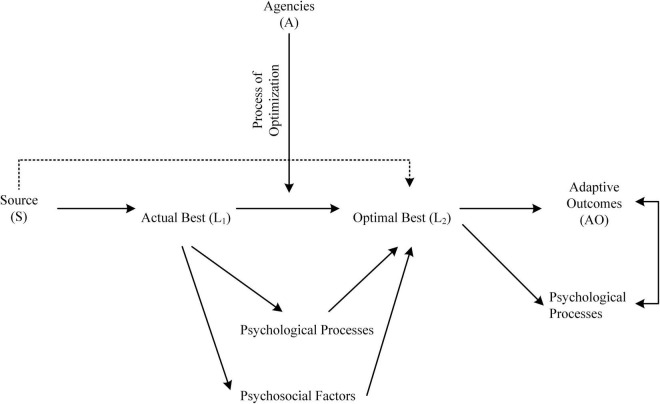
Inquiries of levels of best practice. Adapted from Phan et al. (2019a).

Any conceptualization (e.g., multifaceted structure of mindfulness: Phan et al., 2020b), for that matter, requires some form of scientific validation, which may involve the use of a robust methodological design (e.g., a longitudinal two-group experimental design). For example, the study of optimal best (Fraillon, 2004; Phan et al., 2016, 2017) has so far consisted of a number of correlational studies (e.g., Phan et al., 2018; Phan and Ngu, 2020, Phan and Ngu, 2021b) that yielded clear and comparable evidence, supporting the elucidation of the nature of best practice (e.g., see Figure 4). Arising from this line of research (e.g., the predictive effect of actual best on optimal best) is an interesting proposition for consideration, namely, a focus on the design and development of an appropriate methodological approach for usage. Coinciding with this consideration, we recently published an article in *Frontiers in Psychology* in which we acknowledge a theoretical concept known as “methodological appropriateness” or a “constructive alignment” between a methodological design and the proposed research question(s) under consideration.

The notion of methodological appropriateness (Phan et al., 2019b) is interesting as it emphasizes the importance in appropriateness of a methodological design, which could accurately and adequately address a research objective. Does a particular methodological design adequately prepare for the testing of and the validation of a research objective or relationship? For example, a focus on the *predictive effect* of personal self-efficacy for academic learning on performance outcome may involve the appropriate use of a non-experimental approach which may consist of Likert-scale measures and correlational analyses (e.g., the use of structural equation modeling; Pajares and Miller, 1994; Liem et al., 2008; Martin et al., 2010). In a similar vein, research pertaining to the effectiveness of *an instructional approach* (e.g., the *unitary approach*) for learning may involve an in-class experimental intervention, X, coupled with the use of academic testing and Likert-scale measures (Ngu et al., 2015a,b, 2018).

The study of optimal best (Fraillon, 2004; Phan et al., 2016), however, has raised some interesting insights into the complexity of measurement and assessment of optimization (Fraillon, 2004; Phan et al., 2017, 2019b). To date, for example, we have undertaken several notable studies, non-experimental in nature, which attempted to elucidate empirical evidence and theoretical understanding into the relationship between actual best, L_1_, and optimal best, L_2_. Figure 4 summarizes our research undertakings *via* means of the use of Likert-scale measures (e.g., the Optimal Outcomes Questionnaire: Phan et al., 2016) with both secondary and university students (e.g., Phan et al., 2019a; Phan and Ngu, 2020, 2021b).

In hindsight and in accordance with existing methodological understanding, the use of Likert-scale measures is advantageous as it allows us to explore and validate positive associations between antecedents of L_1_ and L_2_, L_1_ and L_2_, and other psychological processes and psychosocial factors (Phan et al., 2018, 2019a; Phan and Ngu, 2021b). We firmly believe, however, that a non-correlational, non-experimental approach is not sufficed, limiting the accuracy in measurement and assessment of optimization. What we have accomplished so far is a confirmation of a number of predictive and explanatory effects (i.e., β). For example, the positive effect of L_1_ onto L_2_ (Phan et al., 2018; Phan and Ngu, 2020), the positive effect of L_1_ onto different types of psychological processes (e.g., effort expenditure; Phan and Ngu, 2021b), and the positive effect of L_2_ onto other adaptive outcomes (e.g., personal well-being; Phan et al., 2019a). The process of optimization, as Phan et al. (2019b) explain, is more than just the equivalency of a positive association, *r*, or a predictive effect, β. In the recent refinement of the theory of optimization (Fraillon, 2004; Phan et al., 2017), for instance, we conceptualized and introduced a psychological concept known as “energy” (Phan et al., 2019b,2020c) which infers a perceived sense of vitality, liveliness, and absorption. How would we accurately and appropriately measure, assess, and/or validate the enactment of energy (Phan et al., 2019b)?

**FIGURE 5:**
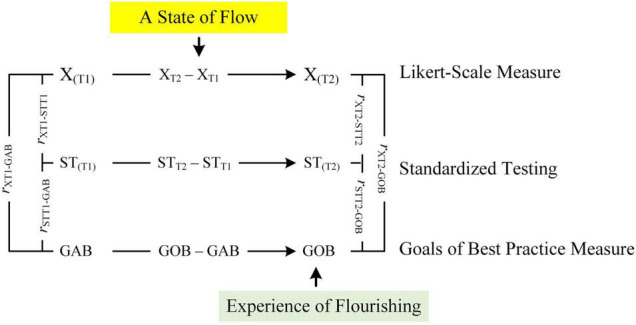
Methodological conceptualization for consideration. T1 = Time 1, T2 = Time 2, r = correlation, GAB = Goal of Actual Best, GOB = Goal of Optimal Best, ST = Standardized testing, and X = Adaptive outcome.

We acknowledge that, at the present stage, there is no adequate methodological design, which could accurately measure and assess the complex nature of optimization. Our recent research development, interestingly, considers the possible use of what we term as a “proxy” methodological design (Phan et al., 2019b). A proxy methodological design, as the term suggests, is an alternative methodology that could produce comparable evidence for inference and explanation of a similar pattern. For example, consider the conceptualization that is shown in Figure 5, in which we adapt from our recent rationalization and reasoning (Phan et al., 2019b). This consideration stipulates a proxy methodological approach which could assist in the measurement and assessment of GsBP, a state of flow, and experience of flourishing.

Figure 5 shows three major interrelated variables that are measured and assessed across two occasions, Time 1 and Time 2: (i) X = an adaptive outcome (e.g., academic engagement); (ii) ST = standardized testing, which may consist of an in-class quiz or a formal exam; and (iii) GAB = goal of actual best and GOB = goal of optimal best (Phan et al., in press). This depiction of a proxy methodological design, importantly, indicates two major propositions: that there is a “within” difference, positive in nature, between each of the three constructs (i.e., X, ST, and GAB and GOB; e.g., X_*T*2_ – X_*T*1_, ST_*T*1_ – ST_*T*2_), and that there is a “between” correspondence, or association, between the three constructs (e.g., *r*_(XT1 – STT1)_, *r*_(STT1 – ABG)_). From this consideration, we propose the following for further research development:

• Evidence of a positive association or positive associations between X, ST, and GAB and GOB may assist us to gauge into the nature of a person’s testament of GAB. A positive association between ST_(T1)_ and GAB, + *r*_(STT1 – GAB)_, and/or a positive association between X_(T1)_ and GAB, + *r*_(XT1 – GAB)_ may provide theoretical insights into the nature of GAB. Particularly, that a modest result in a secondary school student’s standardized test in Calculus at Time 1, for example, may suggest that his GAB is relatively “low key” or modest in nature. In a similar vein, a positive association between X_(T2)_ and GOB, *r*_(XT2 – GOB)_ would provide theoretical understanding into the nature of GOB. For example, a university student’s indication of proactive engagement (i.e., X) in Psychology subject may suggest interesting GsOB.
• Evidence of within positive changes for X (e.g., + Δ X_*T*2_ – X_*T*1_) and/or ST (e.g., + Δ ST_*T*2_ – ST_*T*1_), between Time 1 and Time 2 could potentially reflect a person’s state of flow in a subject matter. A decline or a negative difference for X between Time 1 and Time 2 (i.e., -Δ X_*T*2_ – X_*T*1_), in contrast, would indicate a lack of flow or, alternatively, a state of procrastination, pessimism, and/or underachievement.
• A high level of X, ST, and/or GOB at Time 2 and in tandem with a positive correlation between the three constructs [e.g., + *r*_(XT2 – STT2)_, *r*_(STT2 – GOB)_, and *r*_(XT2 – GOB)_] would indicate evidence of a person’s experience of flourishing in a subject matter.

### In Summary: Issues for Consideration

In summary, the above description provides a thorough analysis of an important inquiry in research development of optimal best, namely, the design of an appropriate methodological approach for implementation which could address a mentioned question and/or aim under consideration. The introduction of the notion or concept of “methodological appropriateness” (Phan et al., 2019b), in this case, has provided theoretical grounding for us to consider, philosophically, a conceptualization of a “proxy” indication of a methodological account for usage. Often the case, some theoretical concepts (e.g., the concept of “Buddhist enlightenment”: Phan et al., 2020b) and relationships (e.g., the enactment of “energy” in the process of optimization: Phan et al., 2019b) are somewhat complex, making it extremely difficult to directly measure and assess. The use of “proxy indicators,” recently described and recommended, is a possibility which could produce valid evidence to support logical explanations of a theoretical concept, relationship between concepts, etc.

We contend that the conceptualization shown in Figure 5 has merits and potentials, providing sound methodological grounding which may assist with the indirect measurement and assessment of GsBP, a state of flow, etc. For example, a student’s limited state of academic engagement in a subject matter may, correspondingly, highlight her GsAB. In a similar vein, a student’s result on a standardized test in Math 101 at Time 2 may reveal some relevant information about the complexity of his GsOB. This conceptualization of a potential proxy methodological design for usage interestingly reflects a non-experimental and correlational approach. It is also plausible, of course, for researchers to refine the conceptualization shown in Figure 5 into a longitudinal methodological design which may incorporate an intervention in between.

An interesting inquiry that coincides with our conceptualization, as shown in Figure 5, involves the use of philosophical psychology (Thagard, 2014, 2018; Phan et al., 2020b) as a proxy methodological approach for usage. This consideration, importantly, is advantageous as it does not involve the use of primary-sourced data and, instead, rely on philosophical beliefs, logical reasoning, and personal intuition for comparison. For example, we recently used Fraillon’s (2004) philosophical reasoning and explanation of optimal best as a basis to advance the development of the theory of optimization (Fraillon, 2004; Phan et al., 2019b) which also led to other related theoretical concepts (e.g., the extent to which *Buddhist mindfulness* could act to optimize a person’s daily functioning; Phan et al., 2020b). In this sense, from our point of view, a researcher’s rationalization of a theoretical concept and/or a relationship between variables may serve as a sound proxy indicator for benchmarking and comparison.

## Practical Implications for Consideration

One significant aspect of the proposed concept of GsBP (Phan et al., in press), aside from theoretical understanding of levels of best practice (e.g., Phan et al., 2016, 2018, 2019b), relates to the importance of practicality, which educators could consider in their teaching and curriculum development. Encouraging GsOB, in this case, is a positive endeavor for consideration. In terms of academic learning, for example, a secondary school student may consider some reflective questions which could relate to her optimal learning experiences (e.g., What do I aspire and/or intend to accomplish within the next 6 months?). This recognition contends that there is credence and positivity in the promotion and encouragement of GsOB, which in turn could motivate individuals to *strive* for ambitious feats and personal successes.

We rationalize that the potential practicality of GsBP (Phan et al., in press) may relate to and involve a positive psychological concept known as “personal striving” or “academic striving” (Phan et al., 2020d; Phan and Ngu, 2020, 2021b). Personal striving, in brief, is defined as “a person’s effortful attempt to seek out a realistic and/or an ambitious endeavor for accomplishment” (Phan et al., 2020d). This concept is positive and intentional, reflecting a person’s internal desire to attain enriched learning experience and/or successful outcome. “I want to strive to achieve optimal best in my study…” is a positive contemplation which may direct and motivate a person to seriously work towards a course of action. We have, to date, undertaken a few correlational studies with consistent evidence, affirming the explanatory power and predictive effect of personal striving. For example, the positive effect of personal striving on effort (β = 0.27, *p* < 0.01) and motivation towards learning (β = 0.22, *p* < 0.01; Phan et al., 2020d), the positive effect of personal striving on effective functioning (β = 0.45, *p* < 0.001), personal resolve (β = 0.43, *p* < 0.001) and actual best (β = 0.21, *p* < 0.01; Phan and Ngu, 2021b), and the positive effect of personal striving on enriched schooling experience (β = 0.12, *p* < 0.01) and academic achievement (β = 0.16, *p* < 0.001; Phan and Ngu, 2020).

The importance of personal striving (e.g., Phan et al., 2020d; Phan and Ngu, 2021b) evidently indicates its potential use as an initiator of GsOB. In this analysis, in terms of practicality, consider the following:

• A first-year university wishing and aspiring to be in the 5% of the class of 2,500 students.
• A bank employee wishing and aspiring to attain an end-of-year promotion.

**FIGURE 6:**
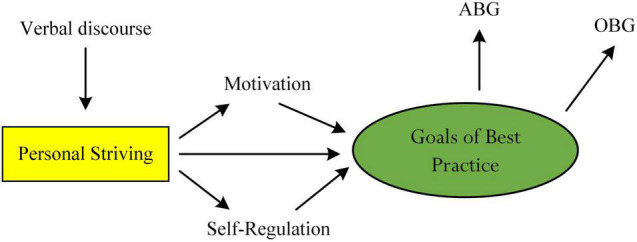
The use of personal striving as educational support.

To facilitate and encourage the mentioned desires and aspirations, consider our proposition as shown in Figure 6 which illustrates the explanatory and predictive role of personal striving (e.g., Phan et al., 2020d; Phan and Ngu, 2021b). The underlying premise of this conceptualization places emphasis on the use of positive verbal discourse (e.g., encouraging feedback; Kim et al., 2010; Pekrun et al., 2014; Gniewosz et al., 2015), which in turn could encourage and promote the development of personal striving. For example, “[…] this pathway is not easy, and you will face obstacles […] but you can do it […]” is a positive statement that pathway is noteworthy for usage, helping to instill persistence, grit, and a perceived sense of personal resolve.

We conceptualize that personal striving, as shown in Figure 6, could act as a direct source of information, governing both motivation and self-regulatory processes (e.g., a person’s evaluation), which in turn would assist a person with their development and setting of GsBP. From our consideration, encouragement of personal striving (e.g., the use of verbal discourse to encourage a student to strive for optimal learning experience in Psychology) may motivate a university student and assist him to self-regulate his learning patterns (e.g., a student may evaluate and monitor their GsOB – for example, is he/she on track?) (Wolters et al., 1996; Zimmerman and Risemberg, 1997; Zimmerman and Schunk, 2001; Schunk and Zimmerman, 2013), resulting in the planning, anticipation, and construction of GsBP.

Best practice (Phan et al., 2018; Ngu et al., 2019) is a performance-related concept that consists of the positive nomenclature of “best.” In this sense, proactive engagement in practice for a subject matter largely reflects a person’s internal desires and aspirations of intent to succeed. We contend that unsuccessful accomplishment of best practice would yield different types of detrimental consequences, such as a state of demotivation, a sense of helplessness, and the feeling of procrastination. In contrast, as evidence has shown (e.g., Phan et al., 2018, 2019a; Phan and Ngu, 2020), successful accomplishment of best practice would associate with and/or predict other related adaptive outcomes (e.g., personal interest in learning). On this basis, academically and non-academically, we recommend the promotion and encouragement of realistic, authentic, and achievable GsBP. In this analysis, the timely setting of GsBP, in tandem with periodic self-regulatory engagements (e.g., the use of monitoring and evaluation – “…. am I on the right track…?), may assist individuals to stay focused, resolved, and motivated to progress from L_1_ onto L_2_.

Interestingly, as one of the reviewers of our earlier draft of the manuscript commented, it is plausible for us to consider mindfulness (Nyanaponika Thera, 1972; Hanh, 1976; Reddy and Roy, 2019; Phan et al., 2020b), in tandem with practice of meditation (Loden, 1996; Bauer-Wu, 2010; Desbordes et al., 2015; Reddy and Roy, 2019), as a source of “intent” and/or motivation for the purpose of assisting one to consider different types of GsOB for accomplishment. For example, does engagement in “walking” meditation motivate a person’s inner intent, resolve, and/or focus, which then could assist with the striving of specific GsOB for accomplishment? This reflective question contends the possibility of a linear trajectory or sequencing in time of relationships between the following: the practice of meditation (e.g., engaging in the walking meditation technique) instills appreciation, feeling, and knowledge of mindfulness which may then heighten the inner motivation and personal resolve of intent, resulting in a person’s clear and strategic vision to construct different types of GsBP for accomplishment. This example in terms of assisting and/or facilitating the active construction of appropriate GsBP, from our point of view, reflects a recent research inquiry into the nature of mindfulness (e.g., Zeidan et al., 2010; Wimmer et al., 2016; Brunner et al., 2017; Malinowski and Shalamanova, 2017; Sevinc et al., 2021) which focuses on cognitive enhancement *via* means of meditation.

## Conclusion

Achieving optimal best or optimal functioning, academically and/or non-academically, is a positive endeavor that is noteworthy for encouragement and promotion. “What is the best that I can do for this subject?” is a personal reflective question that may intricately relate to a student’s internal state of volition, motivation, personal resolve, and self-determination. Our personal interest in this matter (e.g., assisting a person to strive for optimal best) has led to our extensive research undertakings, both conceptually and empirically. Conceptually, for example, we recently developed and proposed a theory (e.g., Phan et al., 2017, 2018, 2019b) which sought to explain the nature of optimization of a person’s achievement of best practice.

Our focus on theoretical understanding of the process of optimization and, more importantly, a person’s achievement of optimal best has, likewise, led to our recent development of a concept which we term as “goals of best practice.” Goals of best practices, as the term connotes, are personal goals that may in effect serve to direct, motivate, and/or facilitate the successful experience and achievement of optimal best. Overall, then, we contend that the proposed concept of GsBP is insightful and may, in fact, feature prominently in the study of positive psychology (Seligman and Csíkszentmihályi, 2000; Seligman et al., 2009). Significant, in this case, is the possibility by which one’s construction and setting of GsOB would partake in a system of change, consisting of a person’s continuing desire, seeking, and motivation to achieve optimal learning and non-learning experiences. Our intention, as detailed throughout, is to present a conceptual analysis of a proposed theoretical model of GsBP, which could provide grounding for further research development. One notable aspect, as discussed in the latter section of the article, is concerned with an inquiry, or inquiries, into the consideration and development of methodological designs for usage. The complexity of optimal best (Fraillon, 2004; Phan et al., 2016, Phan et al., 2019b) and GsBP (Phan et al., in press) makes it somewhat difficult to ascertain accurate and sound empirical evidence, which could elucidate and/or confirm the nature of these concepts. On this basis, our acknowledgment and proposition of the potential use of a “proxy” methodological indicator is noteworthy for consideration.

## Author Contributions

HP and BN contributed equally in the conceptualization, articulation, and writeup of this manuscript. Both authors contributed to the article and approved the submitted version.

## Conflict of Interest

The authors declare that the research was conducted in the absence of any commercial or financial relationships that could be construed as a potential conflict of interest.

## Publisher’s Note

All claims expressed in this article are solely those of the authors and do not necessarily represent those of their affiliated organizations, or those of the publisher, the editors and the reviewers. Any product that may be evaluated in this article, or claim that may be made by its manufacturer, is not guaranteed or endorsed by the publisher.

## Footnotes

1
https://bethesda.net/en/game/doom

2
https://www.dailyscandinavian.com/a-copenhagen-furniture-making-collective/
